# Clinical Utility of Medical Exome Sequencing: Expanded Carrier Screening for Patients Seeking Assisted Reproductive Technology in China

**DOI:** 10.3389/fgene.2022.943058

**Published:** 2022-08-22

**Authors:** Keya Tong, Wenbin He, Yao He, Xiurong Li, Liang Hu, Hao Hu, Guangxiu Lu, Ge Lin, Chang Dong, Victor Wei Zhang, Juan Du, Dongyun Liu

**Affiliations:** ^1^ Center for Reproductive Medicine, Women and Children’s Hospital of Chongqing Medical University, Chongqing, China; ^2^ Chongqing Key Laboratory of Human Embryo Engineering, Women and Children’s Hospital of Chongqing Medical University, Chongqing, China; ^3^ National Engineering and Research Center of Human Stem Cells, Changsha, China; ^4^ School of Basic Medical Science, Institute of Reproductive and Stem Cell Engineering, Central South University, Changsha, China; ^5^ Genetics Centre, Reproductive and Genetic Hospital of CITIC-Xiangya, Changsha, China; ^6^ Chongqing Clinical Research Center for Reproductive Medicine, Women and Children’s Hospital of Chongqing Medical University, Chongqing, China; ^7^ AmCare Genomics Lab, Guangzhou, China

**Keywords:** expanded carrier screening, Han Chinese ethnicity, assisted reproductive technology, preimplantation genetic testing, clinical utility

## Abstract

**Purpose:** Expanded carrier screening (ECS) is an effective method to identify at-risk couples (ARCs) and avoid birth defects. This study aimed to reveal the carrier spectrum in the Chinese population and to delineate an expanded carrier gene panel suitable in China.

**Methods:** Medical exome sequencing (MES), including 4,158 disease-causing genes, was offered to couples at two reproductive centers. It was initially used as a diagnostic yield for potential patients and then used for ECS. Clinical information and ECS results were retrospectively collected.

**Results:** A total of 2,234 couples, representing 4,468 individuals, underwent MES. In total, 254 individuals showed genetic disease symptoms, and 56 of them were diagnosed with genetic diseases by MES. Overall, 94.5% of them were carriers of at least one disease-causing variant. The most prevalent genes were *GJB2* for autosomal recessive disorders and *G6PD* for X-linked diseases. The ARC rate was 9.80%, and couples were inclined to undergo preimplantation genetic testing when diseases were classified as “profound” or “severe.”

**Conclusion:** This study provided insight to establish a suitable ECS gene panel for the Chinese population. Disease severity significantly influenced reproductive decision-making. The results highlighted the importance of conducting ECS for couples before undergoing assisted reproductive technology.

## 1 Introduction

In the past few decades, the development of sequencing technology and the increased awareness of rare inherited diseases have led to the elucidation of a significant number of hereditary diseases. The Online Mendelian Inheritance in Man (OMIM) database has recorded more than 8,000 monogenic diseases to date. In mainland China, the incidence rate of birth defects is approximately 5.6%, and genetic factors account for 40–50% of them. (NHC, 2018)

The objective of carrier screening is to identify asymptomatic individuals who are carrying heterozygous disease-causing variants to avoid the possibility of conceiving offspring with birth defects. It was first proposed to screen Tay–Sachs disease carriers in the Ashkenazi Jewish population. (Kaback, 2000) The strategies for carrier screening markedly reduced the incidence of certain diseases in at-risk populations. (Antonarakis, 2019) The emergence of next-generation sequencing (NGS) has made it possible to simultaneously detect thousands of conditions at an affordable cost and rapid turnaround time. (Tucker et al., 2009) Benefiting from high-throughput sequencing, carrier screening has transitioned from limited ethnicity to general population implementation and from a few diseases to multiple heritable disorders. In 2011, Bell et al. (2011) screened 448 selected severe recessive childhood diseases and found an average genomic carrier burden of 2.8%, and it was the first time NGS was utilized in carrier screening.

Multiple facilities conducted ECS using designed panels for different populations, such as early pregnancy women, ART patients, or the general population. Martin et al. 2015 tested 549 autosomal recessive and X-linked genes for ART-seeking couples in Spain and found that 84% of these individuals were carriers of at least one condition. The investigator estimated that carrier screening prevented 1.25% of birth defects. Franasiak et al. (2016) found that the ECS outcomes affected reproductive decisions in 0.21% of cases after screening 117 conditions in patients in an infertility care center. Haquel et al. (2016) screened 417 pathogenic (P) variants in 94 genes and estimated that the risk of having an autosomal recessive disorder-affected child was at a minimum of 1 in 628 pregnancies in Northern European couples.

In 2015, the American College of Medical Genetics (ACMG) along with other professional societies launched a joint statement focused on principles of expanded carrier screening (ECS) for prenatal or preconception and criteria for panel design. (Edwards et al., 2015) Afterward, the American College of Obstetricians and Gynecologists (ACOG) published recommendations, suggesting that conditions suitable for the ECS panel should meet the following criteria: 1) carrier frequencies equal to or greater than 1/100, 2) clear and definite genotype–phenotype relationships, 3) early onset in life, 4) shortened lifespan, 5) cognitive or physical disability that affects quality of life, and 6) surgical or medical intervention requested. In addition, the conditions included in ECS should be accessed for prenatal diagnosis, and early intervention should be affordable and improve infant outcomes. (Opinion No, 2017) In the latest published ACMG recommendations in 2021, Gregg et al. (2021) proposed 113 autosomal recessive and X-linked genes that were appropriate for ECS. The recommendations have taken into consideration autosomal recessive carrier frequencies equal to or greater than 1/200 and X-linked conditions.

Although the ACMG and the ACOG have made recommendations and guidelines for carrier screening, it is noticed that such announcements are current conclusions drawn from the population in the United States and Europe, rather than extending the investigation to other countries. In particular, carrier screening studies that focused on the Chinese population are limited. The carrier frequencies and ARC rates detected in ECS highly depend on the panel selection. Guo and Gregg (2019) used an exome sequencing database to estimate the carrier frequencies in six different ancestries for 415 genes associated with severe hereditary diseases. They found that the cumulative carrier rates (CCR) were up to 62.9% in the Ashkenazi Jewish population while only 32.6% in the East Asian population. The result implied that the current expanded carrier sequencing panel cannot fully cover the variant spectrum in the East Asian population.

This study aimed to reveal the carrier spectrum in the Chinese population and delineate a gene set suitable for ECS in China. The study retrospectively analyzed the results of medical exome sequencing (MES) for couples seeking advice at reproductive centers.

## 2 Materials and Methods

### 2.1 Study Design

**FIGURE 1:**
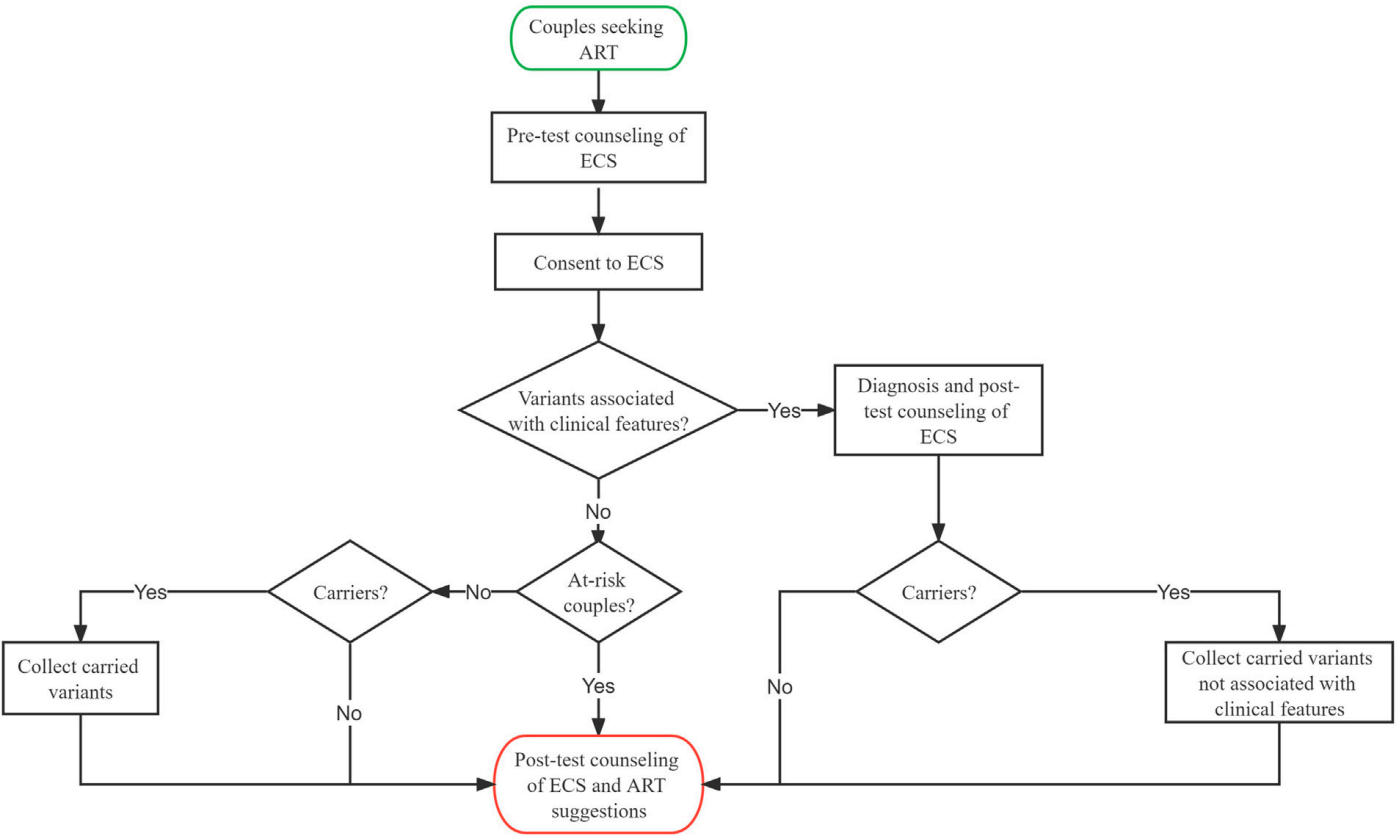
| Clinical workflow of expanded carrier screening in the reproductive center.

As presented in Figure 1, we retrospectively gathered couples from reproductive medicine centers at the Chongqing Health Center for Women and Children and the Genetics Center of Reproductive and Genetic Hospital in CITIC-Xiangya between January 2019 and June 2021. The clinical features of the couples were assessed, and necessary examinations were performed by physicians. ECS was offered to couples who were planning to undergo assisted reproductive technology (ART) or seeking preconception genetic counseling. In particular, MES was first used to identify the variants related to genetic disorders for patients, followed by ECS. The usage and possible outcomes of ECS were explained by physicians to patients before the test. The couples self-reported their age, ethnicity, family history, and gestational history. The following sequencing results were reported to the couples: 1) variants that can explain one’s clinical symptoms (if detected), 2) at-risk couples (ARCs) (see definition in Section 2.2), and 3) carrier status and secondary findings (SFs) (if detected). Post-test counseling was offered to all couples, and ARCs would receive additional ART recommendations from their physicians. Information on medical history, clinical features, ECS results, and post-test interventions were collected. To estimate carrier frequency, P and likely pathogenic (LP) variants from each individual were collected, whereas variants associated with clinical features were excluded from the analysis. Informed consent was signed by all patients.

### 2.2 At-Risk Couple Identification

At-risk couple is defined as follows: both partners carrying a P or LP variant of the same autosomal recessive gene, the female partner carrying a P or LP variant in an X-linked gene, one of the partners carrying a P or LP variant while the other is carrying a variant of the same gene but classified as variant of uncertain significance (VUS), or both partners carrying a VUS of the same gene. The family history of the couples was carefully reviewed. If the proband was available, Sanger sequencing validation was performed on the proband, and the couples were considered ARC when the identified variants segregated in a Mendelian manner. In contrast, if no proband was available, software prediction was used to predict the structure, conservation domain, and function domain for VUS. When the prediction results indicated that the VUS may compromise protein function, the couples were also considered as ARCs.

There are several reasons indicated for ECS, and screened couples were divided into six subgroups: 1) infertility, couples are unable to get pregnant naturally; 2) recurrent miscarriage/stillborn, couples experienced recurrent miscarriage or stillborn; 3) birth defect/ultrasound anomaly, couples gave birth to affected children of known or suspicious genetic diseases or in whom fetal abnormalities were found in prenatal ultrasound; 4) patients, one or both of the partners had clinical manifestations suspected to be genetic diseases; 5) consanguineous marriage, couples were lineal or collateral blood relatives within three generations; and 6) routine screening, both partners were healthy individuals with no family history.

### 2.3 Medical Exome Sequencing

We utilized custom-designed NimbleGen SeqCap probes (Roche NimbleGen, Madison, WI, United States) for in-solution hybridization to enrich target sequences. Target genes included 4,158 genes collected from the OMIM database (updated on June 2021) with definite corresponding diseases, comprising AR, X-linked, and AD genes. Only genes with well-defined genotype–phenotype relationships were included in the panel. The sequencing covered coding exons, and known P variants reported in deep introns or non-coding regions were also included. In terms of gene inclusion, this is the largest carrier screening panel for ECS to date.

### 2.4 Next-Generation Sequencing and Data Analysis

Genomic DNA was extracted from peripheral blood using the Solpure Blood DNA Kit (Magen). To augment target sequences, we used custom-designed NimbleGen SeqCap probes (Roche NimbleGen, Madison, WI, United States) for in-solution hybridization. DNA samples were indexed and sequenced on the AmCareSeq 2000 (Amcare, Guangzhou, China). The average coverage depth was about 200× with over 98% of the target regions covered by at least 20 reads. The sequenced reads were compared with the reference human genome version (GRCh37/hg19). Nucleotide changes found in aligned reads were pulled and analyzed using the NextGENe software (Version 2.4.2) (SoftGenetics, State College, PA, United States). Online software programs PolyPhen-2, SIFT, PROVEAN, MutationTaster, and GeneSplicer were used for *in silico* analysis. The software programs and their corresponding cutoff values were as follows: SIFT (score < 0.05, deleterious; score ≥ 0.05, tolerated), PolyPhen-2 (score ≥ 0.909, probably damaging; score: 0.447–0.909, possibly damaging; score ≤ 0.446, B: benign), PROVEAN (score:−14–2.5, deleterious; score:−2.5–14, neutral), and MutationTaster (A: disease-causing automatic, D: disease-causing, N: polymorphis, P: polymorphism automatic).

Population and literature databases, including gnomAD r2.0.2 (http://gnomad.broadinstitute.org), ClinVar (https://www.ncbi.nlm.nih.gov/clinvar), and OMIM (https://omim.org/), were used to annotate variants. Variants were classified as “P,” “LP,” “VUS,” “likely benign (LB),” or “Benign (B)” according to the ACMG guidelines. (Richards et al., 2015; Riggs et al., 2020)

The Shapiro–Wilk test was utilized for data distribution. Continuous variables were expressed as mean ± SD. Comparison of numeric variables was performed using Student’s *t*-tests. A chi-squared test was used to compare categorical variables and rates. A *p*-value < 0.05 was considered statistically significant.

## 3 Results

### 3.1 Population Demographics

A total of 2,234 couples (4,468 individuals) underwent ECS. They were divided into six subgroups as described in Section 2.2. As shown in Table 1, the average age of men was 33.13 ± 5.37 years, and the average age of women was 31.38 ± 4.72 years. Among them, 94.9% were positive carriers of at least one condition. In addition, Supplementary Table S1 shows that patients carrying two or three variants account for 44.8% of the population, whereas only three (0.13%) men carried a maximum of 10 variants. There were no statistical differences in the carrier rate between women and men (*p* = 0.88).

**TABLE 1:** Demographic and carrier status of 4,468 individuals.

Subgroup	Screened	Percentage (%)	Age		Carrier Status		ARC rate
	n		Female	Male	Positive	Negative	
All	4,468	100	31.4 ± 4.7	33.1 ± 5.7	4,242	226	9.80%
Recurrent miscarriage/stillborn	1756	39	32.2 ± 4.6	34.2 ± 5.4	1,653	103	6.49%
Infertility	1,174	26	29.6 ± 4.0	31.3 ± 5.1	1,119	55	9.71%
Birth defect/ultrasound anomaly	894	20	31.6 ± 4.8	33.5 ± 5.0	852	42	10.24%
Patients	254	6	28.4 ± 5.8	31.7 ± 6.1	239	15	15.66%
Routine screening	340	8	31.2 ± 4.7	31.5 ± 4.2	331	9	8.24%
Consanguineous marriage	50	1	25.3 ± 2.1	26.7 ± 3.2	48	2	32.00%

### 3.2 Patients

Of the 254 individuals in the patient subgroup, 56 were diagnosed as having genetic diseases. The result showed that 22% of the patients were diagnosed as having genetic diseases by MES. The majority of the diagnosed patients were women (82.14%). The most common clinical characteristics were hearing impairment (including hearing loss, deafness, and surdimutism), intellectual disability (ID), eye diseases, and skeletal system diseases. The patients’ information is shown in Supplementary Table S2 **.**

### 3.3 Carrier Frequencies and Recurrent Variants

A total of 1833 A genes and 44 X-linked genes were detected in ECS, among which 1435 and 17 were recurrent, respectively (Supplementary Table S3, S4). The top 10 A genes in all individuals and the top 3 X-linked genes in women are shown in Table 2. The most common disease carried by individuals was *GJB2* (OMIM: 121,011), with a 20.17% carrier frequency, which is associated with *GJB2*-related hearing loss. The most frequent X-linked genes carried by women were *G6PD* (OMIM: 305,900), *DMD* (OMIM: 300,377), and *CACNA1F* (OMIM: 300,110). The top three recurrent variants were 109G > A (p.V37I) of *GJB2* with a frequency of 1 in 6, c.1210–11T > G of *CFTR* with a frequency of 1 in 13, and c.115+6T > C of *IL36RN* with a frequency of 1 in 35 (Table 3).

**TABLE 2:** Carrier frequencies of at-risk and X-linked genes.

Inheritance	Gene	Associated disease	Positive case	Carrier frequency (%)
AR	*GJB2*	*GJB2*-related hearing loss	901	20.17
	*CFTR*	Cystic fibrosis	455	10.18
	*DUOX2*	Thyroid dyshormonogenesis 6	321	7.18
	*SERPINB7*	Palmoplantar keratoderma, nagashima type	167	3.74
	*IL36RN*	Pustular psoriasis, generalized	127	2.84
	*GALC*	Krabbe disease	94	2.10
	*CD36*	Platelet glycoprotein IV deficiency	84	1.88
	*HBB*	ß-Thalassemia	81	1.81
	*USH2A*	Usher syndrome type 2A	77	1.72
	*MYORG*	Idiopathic basal ganglia calcification-7	74	1.66
	*G6PD*	Glucose 6 phosphate dehydrogenase deficiency	40	0.90
XL	*DMD*	Duchenne muscular dystrophy	10	0.22
	*CACNA1F*	Congenital stationary night blindness, type 2A	5	0.11

Note: AR, autosomal recessive; XL, X-linked.

**TABLE 3:** Top 10 recurrent variants detected in expanded carrier screening (ECS).

Gene	Variant location	Recurrent variant	Allele count (N)	1 in _
*GJB2*	NM_004,004.6	c.109G > A (p.V37I)	797	6
*CFTR*	NM_000,492.4	c.1210-11T > G	352	13
*IL36RN*	NM_012,275.3	c.115+6T > C	126	35
*SERPINB7*	NM_003,784.4	c.796C > T (p.R266*)	123	36
*DUOX2*	NM_001363711.2	c.1588A > T (p.K530*)	121	37
*GALC*	NM_000,153.4	c.1901T > C (p.L634S)	91	49
*MYORG*	NM_020,702.5	c.40dupC	74	60
*GJB2*	NM_004,004.6	c.235delC	73	61
*DUOX2*	NM_001363711.2	c.2654G > T	72	62
*C9*	NM_001,737.5	c.346C > T	70	64

### 3.3 Secondary Findings

Different from the primary findings that were relevant to the diagnostic indication, the SFs in exome and genome sequencing were defined as P or LP variants that were not related to the patients’ clinical characteristics. Such findings may not contribute to diagnostic yield but have the potential medical value for patient care. In this study, we found a total of 265 individuals who had positive SFs (Supplementary Table S5). The most frequent SF genes are listed in Table 4: *TTN* (OMIM: 188,840) accounts for 9.56% of positive cases, related to dilated cardiomyopathy. All of the listed genes are autosomal dominant.

**TABLE 4:** Secondary findings detected in ECS.

Gene	Inheritance	Associated disease	Positive case	Percentage (%)
				Total positive *N* = 272 (%)
Top 10 genes				
*TTN*	AD	Dilated cardiomyopathy-1G	26	9.56
*PDE11A*	AD	Primary pigmented nodular adrenocortical disease-2	10	3.68
*MYH6*	AD	Dilated cardiomyopathy-1EE, familial hypertrophic cardiomyopathy-14	8	2.94
*LAMA4*	AD	Dilated cardiomyopathy-1JJ	6	2.21
*ABCA7*	AD	Susceptibility to late-onset Alzheimer’s disease-9	5	1.84
*COMP*	AD	Multiple epiphyseal dysplasia-1	5	1.84
*SLC39A5*	AD	Myopia-24	5	1.84
*SYNE2*	AD	Emery–Dreifuss muscular dystrophy-5	5	1.84
*TUBB1*	AD	Macrothrombocytopenia	5	1.84
*FLNC*	AD	Dilated cardiomyopathy	5	1.84
ACMG recommended secondary finding genes				
*TTN*	AD	TTN-related myopathies	26	9.56
*FLNC*	AD	Dilated cardiomyopathy	5	1.84
*TMEM127*	AD	Hereditary paraganglioma–pheochromocytoma syndrome	1	0.37
*BRCA2*	AD	Hereditary breast and/or ovarian cancer	1	0.37

Note: AD, autosomal dominant.

ACMG launched a 73-gene list for reporting SFs in clinical exome and whole-genome sequencing. (Miller et al., 2021) In the SFs, 4 ACMG SF genes were detected, including 26 cases for *TTN*, 5 cases for *FLNC*, 1 case for *TMEM127* (OMIM: 613,403), and 1 case for *BRCA2* (OMIM: 600,185).

### 3.4 At-Risk Couples

Through ECS, 219 (9.80%) ARCs with 87 different diseases out of 2,234 couples were identified. The comprehensive characteristics of ECS are shown in Table 5. There were 204 couples that carried the same autosomal recessive gene, and the female partner in 1 couple carried an X-linked gene; another 15 couples carried two at-risk genes. Couples in the patient subgroup who were diagnosed with genetic diseases were not considered as ARCs, unless they satisfied the ARC definition (Section 2.2). In addition, among the 219 ARCs, 17 carried at least 1 VUS. PGT was an option offered for these couples. At-risk genes and their related diseases were classified into four groups, 1) profound, 2) severe, 3) moderate, and 4) mild, using a disease severity classification method developed by Lazarin et al. (2014) (Supplementary Table S6). The classifications of the ARCs in each group were as follows: profound (*n* = 13 pairs), severe (*n* = 93 pairs), moderate (*n* = 100 pairs), and mild (*n* = 13 pairs). Furthermore, we compared the ARCs’ attitude towards taking measures between profound/severe and moderate/mild groups. As shown in Table 6, a total of 71 ARCs decided to take measures to specifically target the at-risk gene. Those ARCs found to carry profound/severe disease were more inclined to take measures to avoid having an affected child, which indicated that the reproductive decisions were significantly affected by disease severity (*p* < 0.001). The measures to prevent birth of an affected child included a plan to undergo PGT-M, gamete donation, and pregnancy termination.

**TABLE 5:** Reasons and circumstances for ECS.

Characteristic	Categories	*N* = 219 (pair)	Percentage (%)
		n (pair)	
Reason for screening	Repeated miscarriage/stillborn	57	26.03
	Infertility	57	26.03
	Patients	13	5.94
	Birth defect/ultrasound anomaly	70	31.96
	Routine screening	14	6.39
	Consanguineous marriage	8	3.65
Disease classification^a^	Profound	13	5.94
	Severe	93	42.47
	Moderate	100	45.66
	Mild	13	5.94
Measures taken for at-risk gene(s)	Yes	71	32.42
	No	143	65.30
	Loss to follow-up	5	2.28
Variants classification	Both P or LP	202	92.24
	Both or one VUS	17	7.76

a If couples carried more than one at-risk genes, the classification was consistent with the higher rank.

Note: ECS, expanded carrier screening; P, pathogenic; LP, likely pathogenic; VUS, variant of uncertain significance.

**TABLE 6:** Disease severity classification and measures taken.

	Taken measures	No measures taken		Total	χ^2^ (*p-*value)
Profound/severe	48 (45.28%)	58 (54.72%)		106	
Moderate/mild	23 (20.35%)	90 (79.65%)		113	15.514 (*p* < 0.001)
Total	71 (32.42%)	148 (67.58%)		219	


The detailed characteristics of ARCs are shown in Supplementary Table S7. *GJB2*-related hearing loss (*n* = 75 pairs), cystic fibrosis (*n* = 15 pairs), thyroid dyshormonogenesis (*n* = 12 pairs), and thalassemia (*n* = 16 pairs) were the most common conditions found in the ARCs, which correspond with the related gene carrier frequencies identified in individuals.

When ARCs were identified, not only P or LP variants with high carrier frequencies but also some complex conditions were detected. For example, ARC 152 had a fetus with hydrocephalus and callosal dysplasia detected by prenatal ultrasound, and they chose pregnancy termination. ECS was performed on the couple before their next conception. The result showed that both partners carried VUS of the *CCDC88C* gene that is related to congenital hydrocephalus. The phenotype caused by the *CDDC88C* variants includes fetal enlarged ventricles due to the accumulation of cerebrospinal fluid, and neurological impairment in live births. Considering the symptoms observed *via* prenatal ultrasound and the disease phenotype, the couple was classified as ARC, and the ECS result was reported to them. They had a healthy live birth after undergoing PGT-M. In parallel, ARC 218 underwent IVF and gave birth to an SMA-affected child previously. ECS was used as a routine test before they underwent ART again. The result revealed that they both carried not only a P variant of *SMN1* but also an LP variant of *STRC*. *STRC* is associated with autosomal recessive deafness-16, which did not show symptoms in their firstborn. They decided to undergo PGT-A and PGT-M that targeted both *SMN1* and *STRC*. ARC 219 also received ECS as a routine test before undergoing ART because of infertility. The female partner suffered from aniridia and bilateral horizontal nystagmus. The ECS results showed that they were classified as ARC for *GJB2*, whereas the female partner had a P variant of autosomal dominant condition *PAX6* related to aniridia. They chose to undergo PGT-M that targeted *PAX6* instead of IVF alone. ARC 204 was previously diagnosed with in-born 21-hydroxylase deficiency, and MLPA confirmed the presence of the *CYP21A2* variant. The couple decided to pursue PGT-M to have a healthy child. Therefore, although they were found to be carriers of *UVSSA* related to a mild condition UV-sensitive syndrome, the PGT-M was targeted against *CYP21A2.*

## 4 Discussion

To search for genes suitable for ECS in the Chinese population, this study retrospectively analyzed the results of MES in 2,234 couples. The results showed that 94.9% of individuals were carriers of at least one P or LP variant related to AR or X-linked genes and revealed the highly prevalent genes and their hotspot variants. Disease severity had a significant impact on reproductive decisions made by ARCs.

The carriers of at least one P or LP variant related to AR or X-linked genes rate was 94.4%, similar to the results reported in 78% postive for being a carrier individuals after screening 728 gene disorder pairs in the United States, (Punj et al., 2018) implying the possibility that most individuals were carriers of disease-causing variants. Expanded carrier screening has been provided in many countries for couples at their preconception or early pregnancy. Previous carrier screening for certain diseases in the risk population has reduced the incidence of genetic disorders such as Tay–Sachs disease in the ASJ Ashkenazi Jewish population. Unlike Europe or the United States, ECS in China was only proposed recently and currently lack guidelines for panel design for the Chinese population and post-test counseling. There were previous studies that attempted to perform ECS in China. Zhao et al. (2019) screened 11 genes related to 12 Mendelian conditions in a diverse number of couples. In parallel, Shi et al. (2021) screened 11 recessive conditions in women at early pregnancy in Hong Kong. These two studies drew similar conclusions that α-thalassemia and ß-thalassemia had a high prevalence in the Chinese population. Both of these studies established the significance of ECS in reducing the risk of genetic diseases in newborns. However, these two studies selected panels with limited conditions, which may miss potential ARCs. Xi et al. (2020) used 201 gene panels for couples seeking ART. They reported the CCR (45.91%) of the 187 autosomal recessive genes, which was lower than that of over 60% of 100 gene panels in the ASJ population. (Guo and Gregg, 2019) The CCR results suggested that their panel may not cover the prevalent genetic diseases in China. Thus, genes suitable for ECS in the Chinese population needed further investigation and selection.

In this study, the most common gene in ECS was *GJB2* associated with *GJB2*-related hearing loss. The hotspot variants included c.109G > A (p.V37I) and c.235delC (p.L79Cfs*3). In mainland China, the incidence of hearing loss in newborns was reported to be between 1% and 3.47%, and genetic factors accounted for 50–60% of the patients. We found the carrier frequency of *GJB2* to be 20.17%, which was much higher than previously reported (15%) in all hearing loss-related genes in the Chinese population. (Kim et al., 2013) Moreover, congenital hypothyroidism with an incidence of 0.04% was considered to be the most common neonatal endocrine disease in Chinese newborns. (Deng et al., 2018) *DUOX2* accounted for 44% of the congenital hypothyroidism patients in newborn screening. (Huang et al., 2021) In this study, the results showed that the carrier frequency of *DUOX2* was 7.18%. In addition, *CD36* associated with platelet glycoprotein IV deficiency and *SERPINB7* associated with Nagashima-type palmoplantar keratoderma have been unreported in China, and their prevalence is unclear. These two genes were classified as moderate and mild phenotypes, respectively, and were not included in most ECS panels. Furthermore, *GALC* associated with Krabbe disease causes ID and early death. (Shin et al., 2016) The incidence varies in ethnic groups ranging from 1 in 100 live births in the Druze population (Rafi et al., 1996) to 2 in 1,000,0000 live births in Japan. (Foss et al., 2013) The prevalence in China remains unclear. This study reported that *GALC* had a rather high carrier frequency of 2.10% in China, along with the hotspot mutation c.1901T > C (p.L634S), which accounted for 96.8% (91/94) of the detected variants. This indicated that although *GALC* was not recommended in the gene set according to the ACMG, it should be included in carrier screening panels for the Chinese population. In addition, it has been reported that *CYP21A2* and *FMR1* have a high prevalence in some populations. (Hernandez-Nieto et al., 2020) Yet, such highly homologous genes and GC-rich regions may easily lead to false-positive or false-negative results in NGS. Screening for these genes should be compensated for by other molecular methods. (Li et al., 2015)

The awareness of SFs is important for disease prevention and early intervention. This study also analyzed SFs uncovered in ECS. *TTN* had a high carrier frequency (8.24%) in the Chinese population, which is associated with dilated cardiomyopathy. *TTN* truncating variants were responsible for about 25% of familial dilated cardiomyopathy and in 18% of sporadic cases. *TTN*-related dilated cardiomyopathy has a high penetrance after 40 years of age (>95%). (Kellermayer et al., 2019) There were no *TTN*-related patients identified possibly because the individuals were in their fertile age, were in their 30s, and did not meet the onset age for dilated cardiomyopathy. Screening for *TTN* allows for the prevention or early diagnosis and therapy of *TTN*-related dilated cardiomyopathy. The SFs were mostly associated with late onset diseases (*MYH6*, *FLNC*, *LAMA4*, *ABCA7*, and *SYNE2*) or clinical heterogeneous phenotypes (*TUBB1*, *SLC39A5*, and *COMP*). The deleterious variants were sporadic without hotspot regions.

The ARC rate in this study was 9.80%. Analysis of the usage of PGT in ARCs showed that the severity of the disease had a significant impact on reproductive decision-making. Couples that carried diseases classified as profound or severe were inclined to take measures compared with couples that carried moderate or mild diseases. The severity classification of genetic diseases was discussed by Lazarin et al. (2014), and they focused on the characteristics of the diseases. Consistent with the carrier frequencies in individuals, 75/219 ARCs were *GJB2* carriers, and 15/75 ARCs were *CFTR* carriers. Despite the high carrier frequency of *GJB2*, only 8 in 75 ARCs underwent PGT-M for the targeted *GJB2* variant. The possible reasons were that despite c.109G > A (p.V37I) being P, this variant is a hypomorph and mostly causes mild to moderate hearing loss with late onset and low penetrance (17%). (Kim et al., 2013) A total of 20 individuals with homozygous c.109G > A (p.V37I) variant were detected in this study, but only 6 of them experienced hearing loss, which increased the penetrance to 30% (Supplementary Table S8). However, a consensus interpretation of the p. V37I variant of *GJB2* was launched by the ClinGen Hearing Loss Expert Panel in 2019; they reviewed case–control studies and functional, computational, allelic, and segregation data regarding the variant. The panel concluded that the p. V37I variant of *GJB*2 is pathogenic for autosomal recessive nonsyndromic hearing loss with variable expressivity and incomplete penetrance. (Shen et al., 2019) A recent study also found that homozygous p. V37I *GJB2* is associated with progressive hearing loss in adults, especially over 60 years old. (Chen et al., 2022) Therefore, knowing the ECS results allowed the couples to consider neonatal hearing screening and avoid the hearing loss-inducing factors in adulthood. (Chai et al., 2015) In parallel, variant c.1210-11T > G, also known as IVS8-5T allele, of *CFTR* is the most common allele worldwide. (Chillón et al., 1995) Many genotype–phenotype studies demonstrated that the IVS8-5T allele is pathogenic for congenital bilateral absence of the vas deferens with incomplete penetrance and is a genetic modifier for CF. (Bombieri et al., 2011) In this study, the IVS8-5T allele was mostly found in men with infertility issues. After explaining the disease risk in the genetic counseling, none of the 15 ARCs underwent PGT-M that targeted *CFTR*. Instead of identifying ARCs after giving birth to an affected offspring, ECS helps determine ARCs during preconception and enables the couples to make alternative reproductive decisions. (Ghiossi et al., 2018) PGT allows for the detection of embryos with genetic disorders before implantation to prevent delivering infants with a birth defect. It is also designed to detect monogenic disorders. The utility of PGT-M relies on the confirmation of P genes, which can be achieved through ECS. (Kuliev and Rechitsky, 2017) In addition, for those ARCs who do not take part in PGT-M as an option, the ECS results still provide them with clues to undergo prenatal diagnosis or early intervention for affected newborns. On the other hand, in clinical practice, the ECS results may be challenging to interpret. For example, limited by the current knowledge of genes and variants, detection of a large number of variants in ECS that are classified as VUS provides uncertain information, and couples may be confused in terms of what steps to follow after receiving this information. For couples in whom one partner carried a P or LP variant while the other carried a VUS, it was a great challenge to decide whether to report a positive result to the patients. The clinical validity of the ECS panel relied on the ARC detection rate. Although the ARC rate of 9.80% was considerably higher than those in previous studies, using such a large-scale panel may pose an excessive information overload to both patients and physicians.

The ideal situation for ECS is to identify carriers to provide information regarding the possibility of having an affected child before conception or during early pregnancy. Taken together with the results in this study, we make the following suggestions: 1) Couples who intend to receive ART should undergo ECS regardless of family history and ethnicity. 2) Current ECS panel design is mostly based on carrier frequency and severity. However, due to the complexity of genotype–phenotype correlations, some highly prevalent variants only cause mild phenotypes. The inclusion of such variants should be evaluated to avoid excessive burden on patients and counseling. 3) Adult-onset conditions with high prevalence and severe phenotypes should be considered and included in the carrier screening panel to prevent birth defects and aid them in early management.

In summary, this study performed clinical exome sequencing on couples seeking ART in China. The results revealed that 94.9% of the individuals were carriers of at least one deleterious variant. We preliminary demonstrated a set of highly prevalent genes along with their hotspot variants, providing insight to further establish a suitable ECS gene panel for the Chinese population. The total ARC rate was 9.80% in this study. PGT was offered to the ARCs. Furthermore, the severity of related diseases had a tremendous influence on fertility planning. The study emphasized the significance of couples receiving ECS before undergoing ART.

## Data Availability

The raw data supporting the conclusions of this article will be made available by the authors, without undue reservation, to any qualified researcher.
