# Supplementary material for: Clinical Utility of Medical Exome Sequencing: Expanded Carrier Screening for Patients Seeking Assisted Reproductive Technology in China
Source: Front Genet. 2022 Aug 22;13:943058. doi: 10.3389/fgene.2022.943058 (PMC9441495; doi:10.3389/fgene.2022.943058)
Supplement: Supplementary file 1 [file DataSheet1.docx]

**Supplementary Table 1 Positive conditions in 5177 genes for 4468 individuals**

| **Positive condition** | **Female** | | **Male** | |
| --- | --- | --- | --- | --- |
|  | **N (Total N=2234)** | **Percentage (%)** | **N (Total N=2234)** | **Percentage (%)** |
| 1 | 384 | 17.19% | 397 | 17.77% |
| 2 | 510 | 22.83% | 510 | 22.83% |
| 3 | 489 | 21.89% | 496 | 22.20% |
| 4 | 373 | 16.70% | 382 | 17.10% |
| 5 | 214 | 9.58% | 178 | 7.97% |
| 6 | 103 | 4.61% | 97 | 4.34% |
| 7 | 30 | 1.34% | 37 | 1.66% |
| 8 | 10 | 0.45% | 17 | 0.76% |
| 9 | 5 | 0.22% | 7 | 0.31% |
| 10 | 0 | 0.00% | 3 | 0.13% |
|  |  |  |  | *p*=0.88 |
| **Total positive cases** | 2118 | 94.81% | 2124 | 95.08% |

**Supplementary Table 2 Characteristics of patients diagnosed by ECS**

| **Categories** | **Screened**  **(N=254)** | **Percentage (%)** |
| --- | --- | --- |
|  | **n** |  |
| **Total** | 56 |  |
| **Sex** |  |  |
| Male | 22 | 39.29% |
| Female | 46 | 82.14% |
| **Main clinical characteristics** |  |  |
| Hearing impairment | 17 | 30.36% |
| Intellectual disability | 15 | 26.79% |
| Eye disease | 6 | 10.71% |
| Skeletal system disease | 5 | 8.93% |
| Others | 13 | 23.21% |
| **Variant type** |  |  |
| SNV | 47 | 83.93% |
| CNV | 8 | 14.29% |
| SNV+CNV | 1 | 1.79% |
| **SNV inheritance** |  |  |
| AD | 23 | 41.07% |
| AR | 21 | 37.50% |
| XL | 3 | 5.36% |

**Supplementary Table 3 Top 100 recurrent AR genes**

| **Gene** | **Positive case** |
| --- | --- |
| *GJB2* | 901 |
| *CFTR* | 455 |
| *DUOX2* | 321 |
| *SERPINB7* | 167 |
| *IL36RN* | 127 |
| *GALC* | 94 |
| *CD36* | 84 |
| *HBB* | 81 |
| *USH2A* | 77 |
| *MYORG* | 74 |
| *OCA2* | 74 |
| *SLC26A4* | 73 |
| *C9* | 71 |
| *SLC25A13* | 66 |
| *RP1L1* | 60 |
| *DMGDH* | 52 |
| *LRP1* | 52 |
| *UGT1A1* | 52 |
| *PAH* | 50 |
| *USP45* | 50 |
| *FMO3* | 48 |
| *SLC22A5* | 47 |
| *DUOXA2* | 46 |
| *FCSK* | 46 |
| *MPZL2* | 46 |
| *HBA1/HBA2* | 44 |
| *SBDS* | 42 |
| *SPATA7* | 40 |
| *ABCC6* | 39 |
| *MMACHC* | 36 |
| *UNC13D* | 36 |
| *BCHE* | 34 |
| *CEP290* | 34 |
| *ROM1* | 34 |
| *TMPRSS15* | 34 |
| *DNAH1* | 31 |
| *ABCG5* | 30 |
| *GPNMB* | 30 |
| *OTOGL* | 30 |
| *SLC45A2* | 30 |
| *TNFRSF13B* | 30 |
| *DNAH9* | 29 |
| *MCPH1* | 29 |
| *NME8* | 29 |
| *SPINK1* | 29 |
| *ABCC2* | 28 |
| *CFAP43* | 28 |
| *NR0B2* | 28 |
| *PKHD1* | 28 |
| *TYR* | 28 |
| *ABCA5* | 26 |
| *ALMS1* | 26 |
| *SUGCT* | 26 |
| *FSIP2* | 25 |
| *IDH3A* | 25 |
| *LRPPRC* | 25 |
| *MYO7A* | 25 |
| *SCN9A* | 25 |
| *TRIOBP* | 25 |
| *TSPYL1* | 25 |
| *AMPD1* | 24 |
| *EPO* | 24 |
| *GPAA1* | 24 |
| *GUF1* | 24 |
| *MAT1A* | 24 |
| *PRSS12* | 24 |
| *SLC12A3* | 24 |
| *SPINK5* | 24 |
| *AKR1C2* | 23 |
| *ATP2B2* | 23 |
| *CEP152* | 23 |
| *CHRNA1* | 23 |
| *DNAH5* | 23 |
| *DYSF* | 23 |
| *PMFBP1* | 23 |
| *ASPM* | 22 |
| *CYP4V2* | 22 |
| *EYS* | 22 |
| *GAA* | 22 |
| *LAMA2* | 22 |
| *LOXHD1* | 22 |
| *MYO3A* | 22 |
| *NEB* | 22 |
| *OTOA* | 22 |
| *SLC36A2* | 22 |
| *AIRE* | 21 |
| *F11* | 21 |
| *HPGD* | 21 |
| *MYO15A* | 21 |
| *VPS13B* | 21 |
| *COCH* | 20 |
| *COL6A3* | 20 |
| *CYP24A1* | 20 |
| *DPYD* | 20 |
| *GNE* | 20 |
| *LZTR1* | 20 |
| *PNKP* | 20 |
| *TG* | 20 |
| *TUB* | 20 |
| *ABCA4* | 19 |

**Supplementary Table 4 XL genes detected in ECS**

| **Gene** | **Positive case** |
| --- | --- |
| *G6PD* | 31 |
| *DMD* | 8 |
| *STS* | 4 |
| *ATP11C* | 3 |
| *CACNA1F* | 3 |
| *CLCN4* | 3 |
| *IDS* | 3 |
| *IKBKG* | 3 |
| *DKC1* | 2 |
| *F8* | 2 |
| *HUWE1* | 2 |
| *MID2* | 2 |
| *NHS* | 2 |
| *PGK1* | 2 |
| *PHKA1* | 2 |
| *TMLHE* | 2 |
| *UBQLN2* | 2 |
| *ADGRG2* | 1 |
| *AIFM1* | 1 |
| *ANOS1* | 1 |
| *CDKL5* | 1 |
| *FLNA* | 1 |
| *HSD17B10* | 1 |
| *IGSF1* | 1 |
| *IQSEC2* | 1 |
| *KDM5C* | 1 |
| *L1CAM* | 1 |
| *MAOA* | 1 |
| *MBTPS2* | 1 |
| *MECP2* | 1 |
| *MTM1* | 1 |
| *NR0B1* | 1 |
| *OCRL* | 1 |
| *OFD1* | 1 |
| *PDHA1* | 1 |
| *PHEX* | 1 |
| *POU3F4* | 1 |
| *SERPINA7* | 1 |
| *SHROOM4* | 1 |
| *SLC16A2* | 1 |
| *SYP* | 1 |
| *TEX11* | 1 |
| *WAS* | 1 |
| *WDR45* | 1 |

**Supplementary Table 5 Positive conditions for secondary findings**

| **Positive condition** | **Number** | **Percentage (%)** |
| --- | --- | --- |
| 1 | 268 | 6.00% |
| 2 or more | 4 | 0.09% |
| None | 4196 | 93.91% |
| **Total** | 4468 | 100.00% |

**Supplementary Table 6 Genes related diseases severity classification**

| **Disease** | **Gene** | **OMIM** | **Severity classification** |
| --- | --- | --- | --- |
| Dubin-Johnson syndrome | *ABCC2* | 601107 | Mild |
| Sitosterolemia 2 | *ABCG5* | 605459 | Mild |
| ALMS1 syndrome | *ALMS1* | 606844 | Severe |
| Familial hypertrophic cardiomyopathy-27 | *ALPK3* | 617608 | Severe |
| Primary microcephaly-5 | *ASPM* | 605481 | Severe |
| Harel-Yoon syndrome; Alpha-thalassemia | *ATAD3A* | 612316 | Severe |
| Bardet-Biedl syndrome 10 | *BBS10* | 610148 | Severe |
| Congenital hydrocephalus-1, HYC1 | *CCDC88C* | 611204 | Severe |
| Platelet glycoprotein IV deficiency | *CD36* | 173510 | Moderate |
| Primary microcephaly-6 | *CENPJ* | 609279 | Severe |
| Bardet-Biedl syndrome 14, Joubert syndrome 5 | *CEP290* | 610142 | Severe |
| Cystic fibrosis | *CFTR* | 602421 | Severe |
| 3-Methylglutaconic aciduria type VII | *CLPB* | 616254 | Severe |
| Congenital hypomyelinating neuropathy-3,lethal congenital contracture syndrome-7 | *CNTNAP1* | 602346 | Profound |
| 3-M syndrome 1 | *CUL7* | 609577 | Profound |
| Methemoglobinemia | *CYB5R3* | 613213 | Profound |
| Spastic paraplegia-28 | *DDHD1* | 614603 | Moderate |
| Adams-Oliver syndrome 2 | *DOCK6* | 614194 | Moderate |
| Developmental and epileptic encephalopathy-23 | *DOCK7* | 615730 | Severe |
| Thyroid dyshormonogenesis 6 | *DUOX2* | 606759 | Severe |
| Short-rib thoracic dysplasia-3 with or without polydactyly | *DYNC2H1* | 603297 | Severe |
| Cerebrooculofacioskeletal syndrome-1,COFS1;Cockayne syndrome B ,CSB;de Sanctis-Cacchione syndrome | *ERCC6* | 609413 | Profound |
| Ellis-van Creveld syndrome | *EVC2* | 607261 | Severe |
| Ichthyosis vulgaris | *FLG* | 135940 | Mild |
| Cryptophthalmos unilateral or bilateral,Fraser syndrome 2 | *FREM2* | 608945 | Severe |
| Glycogen storage disease Ia | *G6PC* | 613742 | Severe |
| G6PD deficiency | *G6PD* | 305900 | Mild |
| Glycogen storage disease II | *GAA* | 606800 | Profound |
| Krabbe disease | *GALC* | 606890 | Profound |
| Glutaricaciduria type I | *GCDH* | 608801 | Moderate |
| Pseudoxanthoma elasticum-like disorder with multiple coagulation factor deficiency,Combined deficiency of vitamin K-dependent clotting factors-1 | *GGCX* | 137167 | Moderate |
| GJB2-related hearing loss, DFNB1A | *GJB2* | 121014 | Moderate |
| GM1-gangliosidosis | *GLB1* | 611458 | Profound |
| Jaberi-Elahi syndrome | *GTPBP2* | 607434 | Severe |
| Alpha-thalassemia | *HBA1/HBA2* | 141850 | Severe |
| Beta-thalassemia | *HBB* | 141900 | Severe |
| Growth retardation, impaired intellectual development, hypotonia, and hepatopathy | *IARS* | 600709 | Severe |
| Pustular psoriasis-14 | *IL36RN* | 605507 | Severe |
| Junctional Epidermolysis bullosa with pyloric stenosis | *ITGB4* | 147557 | Severe |
| Amelogenesis imperfecta type IH | *ITGB6* | 147558 | Mild |
| Intellectual developmental disorder Claes-Jensen type | *KDM5C* | 314690 | Severe |
| Orofaciodigital syndrome XV,Joubert syndrome 38 | *KIAA0753* | 617112 | Severe |
| Junctional epidermolysis bullosa | *LAMA3* | 600805 | Severe |
| Combined oxidative phosphorylation deficiency; Spastic ataxia 3 | *MARS2* | 609728 | Severe |
| 3-Methylcrotonyl-CoA carboxylase 2 deficiency | *MCCC2* | 609014 | Severe |
| Bardet-Biedl syndrome 13, Joubert syndrome 28, Meckel syndrome 1 | *MKS1* | 609883 | Profound |
| Combined methylmalonic aciduria and homocystinuria type cblC | *MMACHC* | 609831 | Profound |
| Mucopolysaccharidosis type IIIB | *NAGLU* | 609701 | Profound |
| Primary ciliary dyskinesia-6 | *NME8* | 607421 | Moderate |
| Tyrosinase-positive oculocutaneous albinism type II | *OCA2* | 611409 | Moderate |
| Joubert syndrome 10, Orofaciodigital syndrome I | *OFD1* | 300170 | Severe |
| 5-Oxoprolinase deficiency | *OPLAH* | 614243 | Profound |
| Primary immunodeficiency-9, IMD9 | *ORAI1* | 610277 | Moderate |
| Autosomal recessive deafness-9 | *OTOF* | 603681 | Moderate |
| Nonsyndromic deafness-84B | *OTOGL* | 614925 | Moderate |
| Phenylketonuria | *PAH* | 612349 | Profound |
| Propionicacidemia | *PCCB* | 232050 | Profound |
| Congenital disorder of glycosylation type I | *PGM1* | 171900 | Severe |
| Muscle glycogenosis | *PHKA1* | 311870 | Mild |
| Polycystic kidney disease 4 | *PKHD1* | 606702 | Severe |
| Spermatogenic failure 31 | *PMFBP1* | 618085 | Mild |
| Nonsyndromic hearing loss-84A | *PTPRQ* | 603317 | Moderate |
| Combined cellular and humoral immune defects with granulomas,Severe combined immunodeficiency B cell-negative | *RAG1* | 179615 | Severe |
| Baller-Gerold syndrome | *RECQL4* | 603780 | Severe |
| RIDDLE syndrome | *RNF168* | 612688 | Moderate |
| Hereditary sensory neuropathy type IID | *SCN9A* | 603415 | Moderate |
| Nagashima-type palmoplantar keratoderma | *SERPINB7* | 603357 | Moderate |
| Primary systemic carnitine deficiency | *SLC22A5* | 603377 | Severe |
| Citrullinemia type II | *SLC25A13* | 603859 | Severe |
| Carnitine-acylcarnitine translocase deficiency | *SLC25A20* | 613698 | Severe |
| Autosomal recessive deafness-4 | *SLC26A4* | 605646 | Moderate |
| Spinal muscular atrophy | *SMN1* | 600354 | Severe |
| Cerebral dysgenesis, neuropathy, ichthyosis, and palmoplantar keratoderma syndrome | *SNAP29* | 604202 | Severe |
| Netherton syndrome | *SPINK5* | 605010 | Moderate |
| Autosomal recessive deafness-16 | *STRC* | 606440 | Moderate |
| Developmental and epileptic encephalopathy 18 | *SZT2* | 615463 | Severe |
| Transient infantile liver failure | *TRMU* | 610230 | Severe |
| Pontocerebellar hypoplasia | *TSEN54* | 608755 | Severe |
| Short-rib thoracic dysplasia 4; Nephronophthisis 12 | *TTC21B* | 612014 | Moderate |
| Kaufman oculocerebrofacial syndrome, KOS | *UBE3B* | 608047 | Moderate |
| Retinitis pigmentosa 39 | *USH2A* | 608400 | Moderate |
| UV-sensitive syndrome 3 | *UVSSA* | 614632 | Mild |
| Spinocerebellar ataxia-4 | *VPS13D* | 608877 | Moderate |
| Spinocerebellar ataxia-22 | *VWA3B* | 614884 | Severe |
| Cranioectodermal dysplasia 2 | *WDR35* | 613602 | Severe |
| Cerebellar ataxia, mental retardation, and dysequilibrium syndrome 2 | *WDR81* | 614218 | Severe |
| Spastic paraplegia 15 | *ZFYVE26* | 612012 | Severe |

**Supplementary Table 7 Genes, disease classification, and decisions after ECS**

| **ARC index** | **At-risk gene** | **Related Disease** | **Disease classification** | **N** | **Actions taken for risk gene(s)** |
| --- | --- | --- | --- | --- | --- |
|  | **All** |  |  | 219 |  |
|  | **Carriers of one risk gene** |  |  | 204 |  |
| 1-75 | *GJB2* | *GJB2*-related hearing loss | Moderate | 75 | 8/75 (3 PGT-A+PGT-M, 4 PGT-M, 1 Termination) |
| 76-90 | *CFTR* | Cystic fibrosis | Severe | 15 | 0/15 |
| 91-102 | *DUOX2* | Thyroid dyshormonogenesis | Severe | 12 | 1/12 (1 PGT-M) |
| 103-113 | *HBA1/HBA2* | Alpha-thalassemia | Severe | 11 | 7/11 (6 PGT-M, 1 gamete donation) |
| 114-118 | *HBB* | Beta-thalassemia | Severe | 5 | 5/5 (5 PGT-M) |
| 119-122 | *SERPINB7* | Nagashima-type palmoplantar keratoderma | Mild | 4 | 1/4 (1 PGT-M) |
| 123-125 | *SMN1* | Spinal muscular atrophy | Severe | 3 | 2/3 (2 PGT-M) |
| 126-128 | *FLG* | Ichthyosis vulgaris | Mild | 3 | 0/3 |
| 129, 130 | *CD36* | Platelet glycoprotein IV deficiency | Moderate | 2 | 2/2 (2 PGT-A+PGT-M) |
| 131, 132 | *EVC2* | Ellis-van Creveld syndrome | Severe | 2 | 2/2 (2 PGT-M) |
| 133, 134 | *GALC* | Krabbe disease | Profound | 2 | 1/2 (1 PGT-A+PGT-M) |
| 135, 136 | *IL36RN* | Pustular psoriasis-14 | Severe | 2 | 0/2 |
| 137, 138 | *SLC22A5* | Primary systemic carnitine deficiency | Severe | 2 | 1/2 (1 PGT-M) |
| 139, 140 | *SLC25A20* | Carnitine-acylcarnitine translocase deficiency | Severe | 2 | 2/2 (2 PGT-M) |
| 141 | *CLPB* | 3-methylglutaconic aciduria type VII | Profound | 1 | 0/1 |
| 142 | *CNTNAP1* | Congenital hypomyelinating neuropathy-3, lethal congenital contracture syndrome-7 | Profound | 1 | 0/1 |
| 143 | *CYB5R3* | Methemoglobinemia | Profound | 1 | 0/1 |
| 144 | *GAA* | Glycogen storage disease II | Profound | 1 | 1/1 (1 PGT-A+PGT-M) |
| 145 | *GLB1* | GM1-gangliosidosis | Profound | 1 | 0/1 |
| 146 | *PCCB* | Propionicacidemia | Profound | 1 | 1/1 (1 PGT-M) |
| 147 | *SNAP29* | Cerebral dysgenesis, neuropathy, ichthyosis, and palmoplantar keratoderma syndrome | Severe | 1 | 0/1 |
| 148 | *ZFYVE26* | Spastic paraplegia 15 | Severe | 1 | 1/1 (1 PGT-M) |
| 149 | *ALPK3* | Familial hypertrophic cardiomyopathy-27 | Severe | 1 | 0/1 |
| 150 | *ASPM* | Primary microcephaly-5 | Severe | 1 | 0/1 |
| 151 | *BBS10* | Bardet-Biedl syndrome 10 | Severe | 1 | 1/1 (1 PGT-A+PGT-M) |
| 152 | *CCDC88C* | congenital hydrocephalus-1 | Severe | 1 | 1/1 (1 PGT-M) |
| 153 | *CEP290* | Bardet-Biedl syndrome 14, Joubert syndrome 5 | Severe | 1 | 1/1 (1 PGT-M) |
| 154 | *DOCK6* | Adams-Oliver syndrome 2 | Severe | 1 | 0/1 |
| 155 | *DOCK7* | Developmental and epileptic encephalopathy-23 | Severe | 1 | 1/1 (1 PGT-M) |
| 156 | *DYNC2H1* | Short-rib thoracic dysplasia-3 with or without polydactyly, SRTD3 | Severe | 1 | 0/1 |
| 157 | *ERCC6* | Cerebrooculofacioskeletal syndrome-1,COFS1;Cockayne syndrome B ,CSB;de Sanctis-Cacchione syndrome | Profound | 1 | 1/1 (1 PGT-SR+PGT-M) |
| 158 | *FREM2* | Cryptophthalmos unilateral or bilateral, Fraser syndrome 2 | Severe | 1 | 1/1 (1 PGT-A+PGT-M) |
| 159 | *GCDH* | Glutaricaciduria type I | Moderate | 1 | 1/1 (1 PGT-A+PGT-M) |
| 160 | *GTPBP2* | Jaberi-Elahi syndrome | Severe | 1 | 1/1 (1 PGT-M) |
| 161 | *IARS* | Growth retardation, impaired intellectual development, hypotonia, and hepatopathy | Severe | 1 | 0/1 |
| 162 | *KDM5C* | Intellectual developmental disorder Claes-Jensen type | Severe | 1 | 1/1 (1 PGT-A+PGT-M) |
| 163 | *MARS2* | Combined oxidative phosphorylation deficiency; Spastic ataxia 3 | Severe | 1 | 1/1 (1 PGT-M) |
| 164 | *MCCC2* | 3-Methylcrotonyl-CoA carboxylase 2 deficiency | Severe | 1 | 0/1 |
| 165 | *MKS1* | Bardet-Biedl syndrome 13, Joubert syndrome 28, Meckel syndrome 1 | Profound | 1 | 1/1 (1 PGT-M) |
| 166 | *MMACHC* | Combined methylmalonic aciduria and homocystinuria type cblC | Profound | 1 | 0/1 |
| 167 | *NAGLU* | Mucopolysaccharidosis type IIIB | Profound | 1 | 0/1 |
| 168 | *PAH* | Phenylketonuria | Profound | 1 | 1/1 (1 PGT-M) |
| 169 | *RECQL4* | Baller-Gerold syndrome | Severe | 1 | 1/1 (1 PGT-A+PGT-M) |
| 170 | *SZT2* | Developmental and epileptic encephalopathy 18 | Severe | 1 | 1/1 (1 PGT-M) |
| 171 | *TSEN54* | Pontocerebellar hypoplasia | Severe | 1 | 1/1 (1 PGT-A+PGT-M) |
| 172 | *UBE3B* | Kaufman oculocerebrofacial syndrome, KOS | Severe | 1 | 0/1 |
| 173 | *WDR35* | Cranioectodermal dysplasia 2 | Severe | 1 | 1/1 (1 PGT-M) |
| 174 | *WDR81* | Cerebellar ataxia, mental retardation, and dysequilibrium syndrome 2 | Severe | 1 | Loss to follow-up |
| 175 | *ALMS1* | ALMS1 syndrome | Severe | 1 | 1/1 (1 PGT-M) |
| 176 | *CUL7* | 3-M syndrome 1 | Moderate | 1 | 1/1 (1 PGT-M) |
| 177 | *DDHD1* | Spastic paraplegia-28 ,SPG28 | Moderate | 1 | 0/1 |
| 178 | *G6PC* | Glycogen storage disease Ia | Severe | 1 | 0/1 |
| 179 | *GGCX* | Pseudoxanthoma elasticum-like disorder with multiple coagulation factor deficiency, Combined deficiency of vitamin K-dependent clotting factors-1 | Moderate | 1 | 0/1 |
| 180 | *ITGB4* | Junctional Epidermolysis bullosa with pyloric stenosis | Severe | 1 | 0/1 |
| 181 | *KIAA0753* | Orofaciodigital syndrome XV,Joubert syndrome 38 | Severe | 1 | 0/1 |
| 182 | *NME8* | Primary ciliary dyskinesia-6 | Moderate | 1 | 0/1 |
| 183 | *OCA2* | Tyrosinase-positive oculocutaneous albinism type II | Moderate | 1 | 1/1 (1 PGT-A+PGT-M) |
| 184 | *ORAI1* | Primary immunodeficiency-9, IMD9 | Moderate | 1 | 0/1 |
| 185 | *OTOF* | Autosomal recessive deafness-9 | Moderate | 1 | 0/1 |
| 186 | *OTOGL* | Nonsyndromic deafness-84B | Moderate | 1 | 1/1 (1 PGT-A+PGT-M) |
| 187 | *PGM1* | Congenital disorder of glycosylation type I | Severe | 1 | 0/1 |
| 188 | *PKHD1* | Polycystic kidney disease 4 | Severe | 1 | 1/1 (1 PGT-M) |
| 189 | *PTPRQ* | Nonsyndromic hearing loss-84A | Moderate | 1 | 1/1 (1 PGT-M) |
| 190 | *RAG1* | Combined cellular and humoral immune defects with granulomas, Severe combined immunodeficiency B cell-negative | Severe | 1 | Loss to follow-up |
| 191 | *RNF168* | RIDDLE syndrome | Moderate | 1 | 1/1 (1 PGT-A+PGT-M) |
| 192 | *SCN9A* | Hereditary sensory neuropathy type IID | Moderate | 1 | 0/1 |
| 193 | *SLC26A4* | Autosomal recessive deafness-4 | Moderate | 1 | 1/1 (1 PGT-A+PGT-M) |
| 194 | *TTC21B* | Short-rib thoracic dysplasia 4, Nephronophthisis 12 | Moderate | 1 | 1/1 (1 PGT-A+PGT-M) |
| 195 | *USH2A* | Retinitis pigmentosa 39 | Moderate | 1 | 1/1 (1 PGT-M) |
| 196 | *ABCC2* | Dubin-Johnson syndrome | Mild | 1 | 0/1 |
| 197 | *ABCG5* | Sitosterolemia 2 | Mild | 1 | 0/1 |
| 198 | *ITGB6* | Amelogenesis imperfecta type IH | Mild | 1 | 0/1 |
| 199 | *LAMA3* | Junctional epidermolysis bullosa | Severe | 1 | 0/1 |
| 200 | *OPLAH* | 5-oxoprolinase deficiency | Mild | 1 | 0/1 |
| 201 | *PMFBP1* | Spermatogenic failure 31 | Mild | 1 | 0/1 |
| 202 | *SPINK5* | Netherton syndrome | Moderate | 1 | 1/1 (1 PGT-M) |
| 203 | *TRMU* | Transient infantile liver failure | Severe | 1 | 1/1 (1 gamete donation) |
| 204 | *UVSSA* | UV-sensitive syndrome 3 | Mild | 1 | 1/1 (1 PGT-M) |
|  | **Carriers of two risk genes** |  |  | 15 |  |
| 205-207 | *GJB2, G6PD* | GJB2-related hearing loss | Moderate | 3 | 0/3 |
|  |  | G6PD deficiency | Mild |  |  |
| 208, 209 | *GJB2, HBA1/HBA2* | GJB2-related hearing loss | Moderate | 2 | 2/2 (1 PGT-M, 1 PGT-A+PGT-M) |
|  |  | Alpha-thalassemia | Severe |  |  |
| 210 | *GJB2, HBB* | GJB2-related hearing loss | Moderate | 1 | 1/1 (1 PGT-M) |
|  |  | Beta-thalassemia | Severe |  |  |
| 211 | *ATAD3A, HBA1/HBA2* | Harel-Yoon syndrome | Severe | 1 | 1/1 (1 PGT-A+PGT-M) |
|  |  | Alpha-thalassemia | Severe |  |  |
| 212 | *CENPJ, VWA3B* | Primary microcephaly-6 | Severe | 1 | 1/1 (1 PGT-M) |
|  |  | Spinocerebellar ataxia-22 | Severe |  |  |
| 213 | *GJB2, OFD1* | GJB2-related hearing loss | Moderate | 1 | 0/1 |
|  |  | Joubert syndrome 10, Orofaciodigital syndrome I | Severe |  |  |
| 214 | *GJB2, PHKA1* | GJB2-related hearing loss | Moderate | 1 | 0/1 |
|  |  | Muscle glycogenosis | Mild |  |  |
| 215 | *GJB2, SLC25A13* | GJB2-related hearing loss | Moderate | 1 | 0/1 |
|  |  | Citrullinemia type II | Severe |  |  |
| 216 | *GJB2, STRC* | GJB2-related hearing loss | Moderate | 1 | 0/1 |
|  |  | Autosomal recessive deafness-16 | Moderate |  |  |
| 217 | *GJB2, VPS13D* | GJB2-related hearing loss | Moderate | 1 | 1/1 (1 PGT-A+PGT-M) |
|  |  | Spinocerebellar ataxia-4 | Moderate |  |  |
| 218 | *SMN1, STRC* | Spinal muscular atrophy | Severe | 1 | 1/1 (1 PGT-A+PGT-M) |
|  |  | Autosomal recessive deafness-16 | Moderate |  |  |
| 219 | *GJB2, PAX6* | GJB2-related hearing loss | Moderate | 1 | 1/1 (1 PGT-M) |
|  |  | Aniridia | Moderate |  |  |

**Note:** PGT, preimplantation genetic testing; PGT-A, PGT for aneuploidies; PGT-M, preimplantation genetic testing for monogenetic conditions; PGT-ST, PGT for structural chromosomal rearrangements.

**Supplementary Table 8 Clinical features of individuals carried homozygous *GJB2* c.109G>A variant**

| Sex | Age | Clinical feature | *GJB2*-related syndrome |
| --- | --- | --- | --- |
| M | 31y | Infertility,low quality of sperm | No |
| M | 26y | None | No |
| F | 42y | Hyperthyreosis,ichthyosis | No |
| M | 31y | Moderate neural hearing loss,bilateral | Yes |
| M | 29y | Allergic to antibiotics | No |
| F | 29y | Prenatal ultrasound showed fetal abnormality | No |
| M | 38y | Hearing loss, diabetes,renal calculus, hyperlipemia | Yes |
| F | Unkown | PCOS (consanguineous marriage) | No |
| M | 40y | Adverse pregnancy outcome of suspicious inherited metabolic disorders | No |
| M | 33y | Adverse pregnancy outcome of suspicious inherited metabolic disorders | No |
| F | 28y | Prenatal ultrasound showed fetal abnormality | No |
| M | 24y | Hearing loss | Yes |
| F | 28y | None | No |
| M | 32y | Mild hearing loss, achromatopsia | Yes |
| F | 23y | Intellectual disability | No |
| M | 28y | None | No |
| F | 28y | Infertility,hypothyroidism, alpha-Thalassemia | No |
| F | 27y | Infertility | No |
| F | 29y | Hearing loss | Yes |
| F | 25y | Hearing loss,intellectual disability | Yes |

M, male; F, female; G, gestation; P, parturition; PCOS, polycystic ovary syndrome
